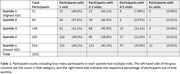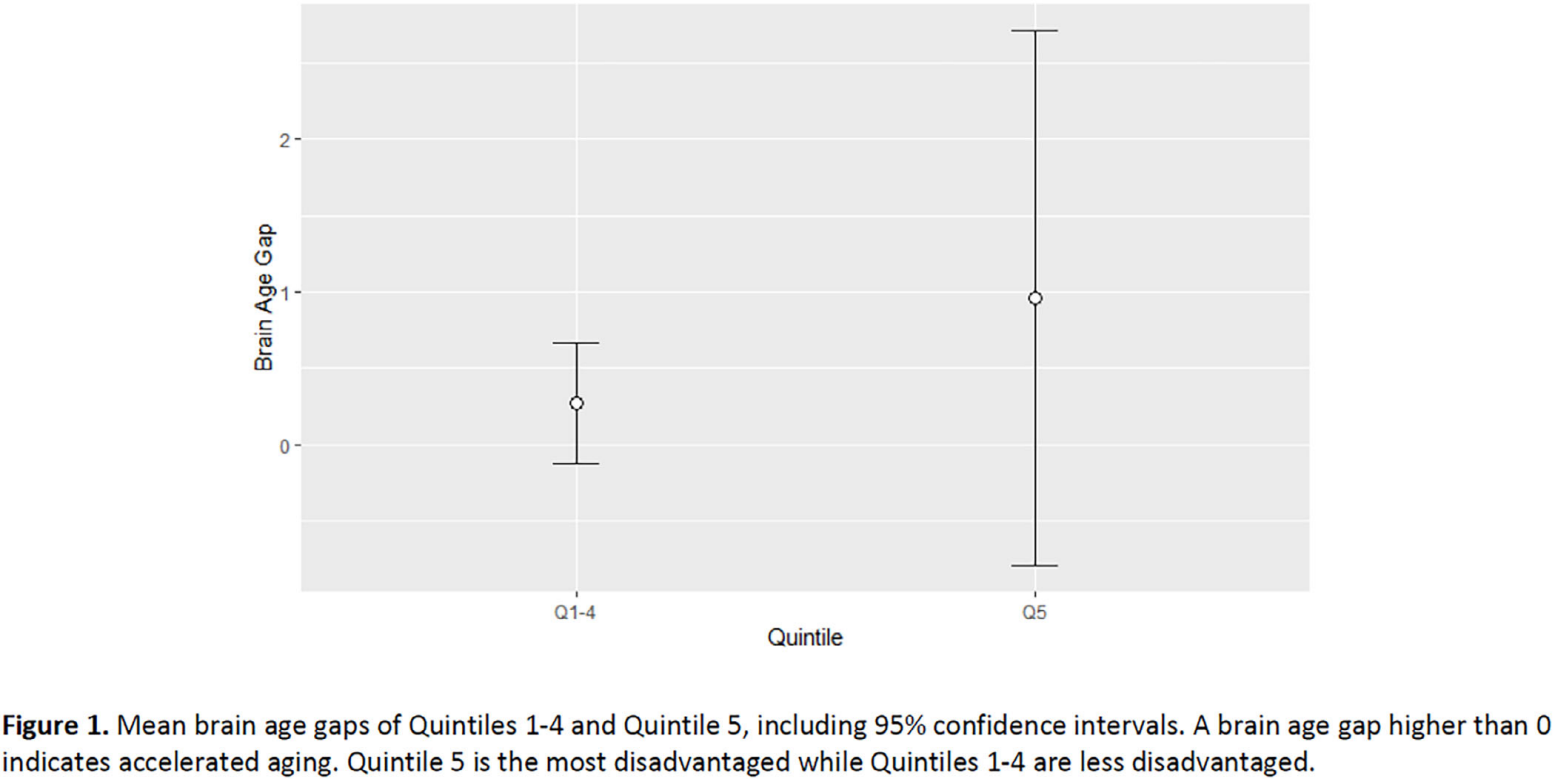# The relationship between neighborhood disadvantage and the brain age gap

**DOI:** 10.1002/alz.092973

**Published:** 2025-01-09

**Authors:** Yolana C Martin, Nagesh Adluru, Veena A. Nair, Vivek Prabhakaran, Andrew L Alexander, Sterling C. Johnson, Sanjay Asthana, W. Ryan Powell, Amy J.H. Kind, Barbara B. Bendlin

**Affiliations:** ^1^ University of Wisconsin‐Madison, Madison, WI USA; ^2^ Center for Health Disparities Research, University of Wisconsin‐Madison, School of Medicine and Public Health, Madison, WI USA; ^3^ Wisconsin Alzheimer's Disease Research Center, School of Medicine and Public Health, University of Wisconsin‐Madison, Madison, WI USA; ^4^ Waisman Laboratory for Brain Imaging and Behavior, University of Wisconsin‐Madison, Madison, WI USA; ^5^ Department of Psychiatry, University of Wisconsin‐Madison, Madison, WI USA; ^6^ Department of Medical Physics, University of Wisconsin‐Madison, Madison, WI USA; ^7^ School of Medicine and Public Health, University of Wisconsin‐Madison, Madison, WI USA; ^8^ Wisconsin Alzheimer's Disease Research Center, Madison, WI USA; ^9^ Department of Medicine, Geriatrics Division, School of Medicine and Public Health, University of Wisconsin‐Madison, Madison, WI USA

## Abstract

**Background:**

Accelerated aging is strongly linked to adverse social exposome and accelerated aging of the brain may be a dementia risk factor. Machine‐learning can estimate the biological “brain age” from neuroimages, which provides complementary information to the chronological/calendar age. The difference between biological and chronological age is referred to as the “brain age gap.” The Area Deprivation Index is a metric representing 17 indicators of neighborhood level poverty, education, employment, and physical environment, and provides a robust measure of the social exposome. The purpose of this work was to test the relationship between neighborhood‐level disadvantage and accelerated brain aging using brain age gap estimates in a Wisconsin cohort.

**Method:**

Brain age was estimated using T1‐weighted MR images from 1052 participants enrolled in the Wisconsin Registry for Alzheimer’s Prevention or Wisconsin Alzheimer’s Disease Research Center studies. Brain age was estimated using a publicly available deep learning model called the two‐stage‐age‐network (TSAN) that was pre‐trained on >4000 scans from OASIS, ADNI‐I, and PAC‐2019 datasets. Average brain age was used when multiple images were available at the same time point. Participants’ addresses were geolinked to their statewide ranking of neighborhood disadvantage using a time‐concordant ADI. Participants were binned by quintile based on state ADI rank, where Quintile 5 comprised of participants with highest ADI (Table 1). In the analyses, Quintile 5 was compared to a grouped category of Quintiles 1‐4. A marginal means test was conducted on a fitted one‐way ANOVA model to assess differences in mean brain age gaps between Quintile 5 and Quintiles 1‐4. The ANOVA model used quintile as a fixed effect predictor variable of brain age gap and a random effect of participant ID to account for participants with multiple visits.

**Result:**

The mean brain age gap for Quintile 5 was 0.96 years (‐0.79, 2.71), and for Quintiles 1‐4 was 0.27 years (‐0.12, 0.66) (Figure 1).

**Conclusion:**

This analysis found that the mean brain age gap does not significantly differ between Quintile 5 and Quintiles 1‐4 (p≤0.45), however statistical power was low given very broad confidence intervals. Future work will test for longitudinal effects and incorporate a larger sample.